# Clinicopathological features of incidentally detected metastatic thyroid papillary carcinoma in cervical lymph nodes of non-thyroid cancer patients: a retrospective analysis of 31cases

**DOI:** 10.1186/s13000-023-01370-4

**Published:** 2023-07-15

**Authors:** Chunfang Hu, Haifeng Zhang, Lixia Chu, Tian Qiu, Haizhen Lu

**Affiliations:** Department of Pathology, National Clinical Research Center for Cancer/Cancer Hospital, National Cancer Center, Chinese Academy of Medical Sciences and Peking Union Medical College, Beijing, China

**Keywords:** Incidental finding, Papillary thyroid cancer, Neck dissections, Metastasis

## Abstract

**Background:**

The incidental finding of thyroid inclusions in lymph nodes of neck dissections of non-thyroid cancer patients is an unusual event. It is still controversial for pathologists about whether this represents benign inclusions or metastatic papillary thyroid carcinoma (PTC). This study is to analyze clinicopathological features of such cases in an attempt to explore their clinical implications.

**Methods:**

Pathological data were searched for incidentally detected PTC of cervical lymph nodes in non-thyroid cancer cases. Clinicopathological characteristics were reevaluated and recorded. BRAF V600E protein expression and sequencing analysis was then performed in cases with sufficient tissues.

**Results:**

31 patients had an incidental finding of PTC in lymph nodes of patients with non-thyroid cancer. BRAF immunohistochemical staining were performed in 17 metastatic lymph nodes with sufficient tumor tissues, and 6 were positive. BRAF V600E point mutation was detected in 5 of 6 BRAF V600E positive cases. Subsequent imaging examinations of the thyroid showed no nodules or calcifications/benign nodules in 20 patients, and suspected malignant nodules in 5 patients. 12 patients underwent total thyroidectomy or ipsilateral lobectomy, and 6 showed PTC in postoperative pathological examinations. The remaining 19 patients without surgery were kept under active surveillance, and no one had recurrence of PTC.

**Conclusion:**

Incidentally discovered PTC in lymph nodes has usually interpreted as metastasis from a clinical occult thyroid primary cancer, but primary PTC was not always detected. This suggests it could be double occult lesions. With regards to concurrence with highly malignant tumor, most patients could keep regular surveillance.

**Supplementary Information:**

The online version contains supplementary material available at 10.1186/s13000-023-01370-4.

## Introduction

Thyroid inclusions may be incidentally discovered in cervical lymph nodes during histological examination of neck dissections performed in nonthyroidal cancer. These inclusions mostly show malignant features of PTC and thus are considered as occult metastatic PTC. However, they may display benign-appearance thyroid follicles in a few cases. There are contradictory opinions for such cases. Some authors believe these thyroid benign-looking follicles are well-differentiated metastatic PTC from primary thyroid carcinoma, but others consider them to be benign thyroid heterotopia [1–3]. In addition to diagnostic dilemma, clinical management on these patients is also controversial. The focus of arguments is whether the clinicians should choose surgery or keep active surveillance. PTC patients usually have a good prognosis even in the presence of lymph nodes metastasis. As the occult metastatic PTC often occurs in the context of more aggressive squamous cell carcinomas, the clinical significance and management of incidentally detected metastatic PTC have not been well defined. Especially when imaging finding is negative, the focus might be extremely small in gross examination, resulting in difficulty in finding the primary thyroid tumor in some cases.

Mutation of the BRAF gene is the most common genetic event in PTC, and it has been found in more than 40% of these tumors [4–6]. BRAF V600E point mutation is accounting for 98% of all BRAF point mutations and plays an important role in the pathogenesis and progression of PTC. BRAF V600E point mutation is reported to be present in only PTC and its congeners, but not in benign thyroid lesions [7]. Therefore, detection of BRAF V600E mutation is of great help for diagnosis of PTC, while immunohistochemical staining of BRAF V600E can be used as an effective substitution for molecular detection [8–11].

In this study, we retrospectively reviewed the pathological database from patients undergoing surgery of neck dissection of non-thyroid carcinomas, analyzed the clinicopathological features of the incidentally detected PTC in cervical lymph nodes, detected BRAF V600E expression and mutations in cases with sufficient tumor tissues, and further tracked their imaging status and postoperative pathology of corresponding thyroid tissues, in an attempt to explore their clinical implications and significance.

## Materials and methods

### Sample collection

The study was approved by the ethics committee and institutional review board of Cancer Hospital, Chinese Academy of Medical Sciences (CHCAMS, 21/517–3188), and all patients were exempt from informed consent. We retrospectively searched the pathological database from patients undergoing surgery of neck dissection in National Cancer Center during the period from 2011 to 2021. 2650 neck dissections were performed in patients with head and neck non-thyroid carcinoma, and 31 patients with an incidental finding of PTC in cervical lymph nodes were identified. Clinicopathological characteristics of these cases including their corresponding cancers were reevaluated and recorded by two senior pathologists. Subsequently, imaging examination and postoperative pathology of primary thyroid were also reviewed and evaluated.

### Immunohistochemical staining (IHC)

Immunohistochemical staining for one or several thyroid related markers (PAX8/TTF1/TG/CK19) was carried out in cases with available tumors to confirm their thyroid origin. BRAF V600E staining was performed in 17 lymph nodes. BRAF V600E (Ventana VE1 Mouse Monoclonal Primary Antibody) IHC staining was performed on 4 μm sections of formalin-fixed, paraffin-embedded tissues by using Ventana Benchmark IHC automated slide strainers in combination with the OptiView DAB IHC detection kit as previously described [12]. BRAF positive staining was defined as ≥ 90% cytoplasmic staining of tumor cells with weak to strong intensity.

### Amplification refractory mutation system (ARMS)

To further detect BRAF mutation, 6 cases with BRAF V600E positive expression were then examined by using amplification refractory mutation system (ARMS). The mutational status of BRAF V600E was detected using the Human BRAF Gene Mutation Detection Kits (ACCB, Beijing, China). The kits applied quantitative real time PCR (qRT-PCR) platform combining ARMS primers and Taq Man probes, and testing was conducted as previously described [13]. Briefly, 5 ng of genomic DNA and PCR master mix were added and proceeded for Real-time PCR analysis which was performed for 5 min at 95 °C, followed by 40 cycles of 95 °C for 15 s, and 60 °C for 1 min. Mutation subtypes were determined according to threshold count (Ct, mutations were identified when the Ct value < 36), following the manufacturer’s instructions.

## Results

31 patients with an incidental finding of PTC in lymph nodes were identified in 2650 patients, indicating a prevalence of 1.2% in this cohort. 27 were male and 4 were female. The median age was 62 (ranging from 39 to 70 years old). 29 cases of primary tumor were squamous cell carcinoma (9 larynx, 6 hypopharynx, 6 esophagus, 4 oral cavity, 2 oropharynx and 2 salivary gland), and 1 was low grade polymorphous adenocarcinoma of oropharynx, and 1 was myoepithelial carcinoma of oral cavity. Regarding the clinical stage of primary tumor, 4 were stage I, 5 stage II, 11 stage III and 11 stage IV (Tables 1 and 2).

**Table 1:** Location of initial non-thyroid carcinoma of head and neck

Location	N(%)
Larynx	9(29%)
Hypopharynx	6(19.4%)
Esophagus	6(19.4%)
Oral cavity	5(16.1%)
Oropharynx	3(9.7%)
Salivary gland	2(6.4%)

**Table 2:** Basic information of enrolled patients

Characteristics	N (%)	
**Age (years)**	62 ± 8.47	
**Sex**		
Male	27(87.1%)	
Female	4(12.9%)	
**Histology of initial carcinomas**		
Squamous cell carcinoma	29(93.5%)	
Low grade adenocarcinoma	1(3.25%)	
Myoepithelial carcinoma	1(3.25%)	
**Clinical stage**		
Stage I	4(12.9%)	
Stage II	5(16.1%)	
Stage III	11(35.5%)	
Stage IV	11(35.5%)	

**Fig. 1:**
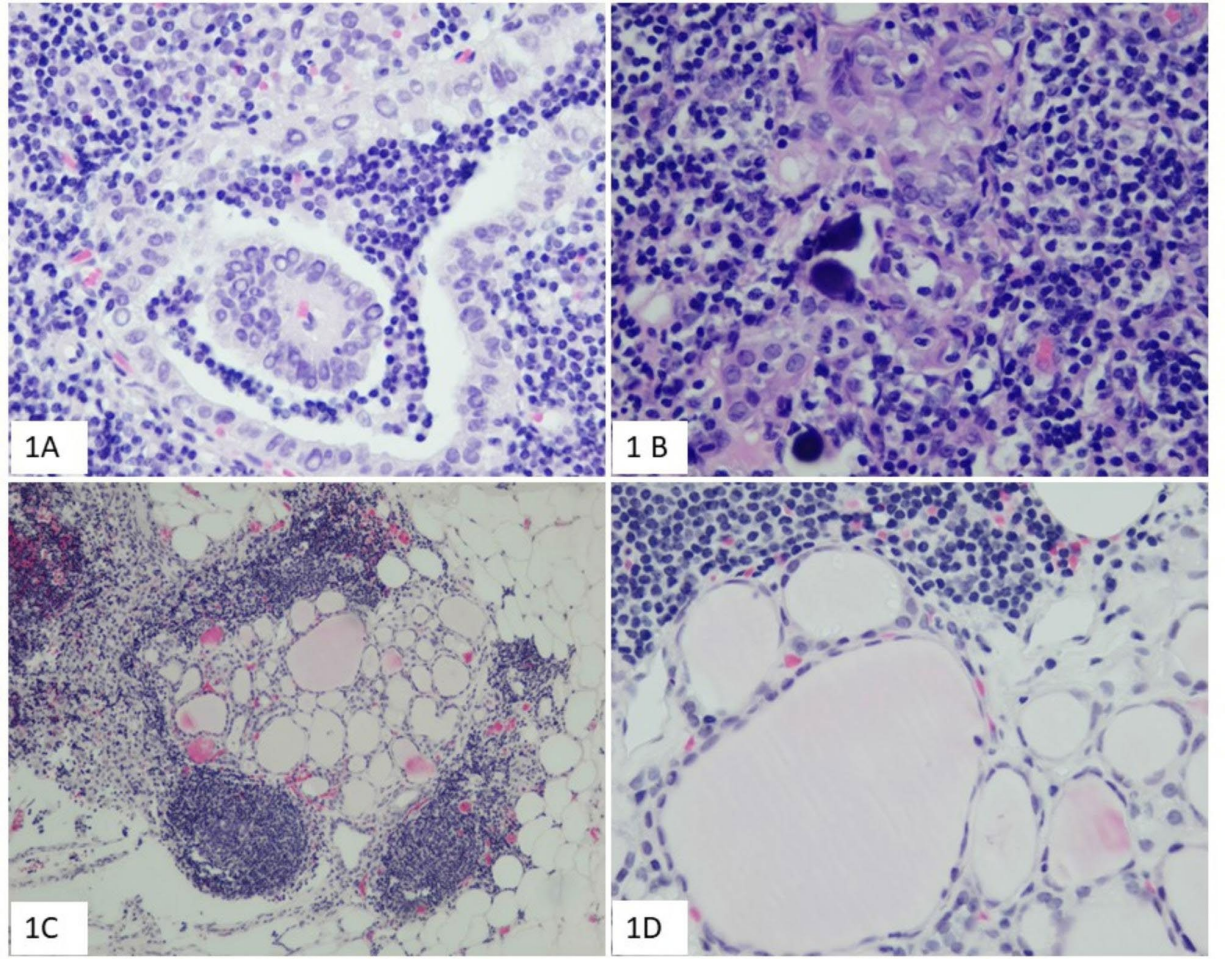
morphology of incidentally detected thyroid papillary carcinoma in cervical lymph nodes. Papillary structure and clear nuclei (1** A** HE×400), Psammoma bodies (1**B** HE×400), bland-looking thyroid follicular structure (1** C** HE×100), thyroid follicles lined by cuboidal epithelium with minimal nuclear atypia in a higher magnification (1**D** HE×400)

**Fig. 2:**
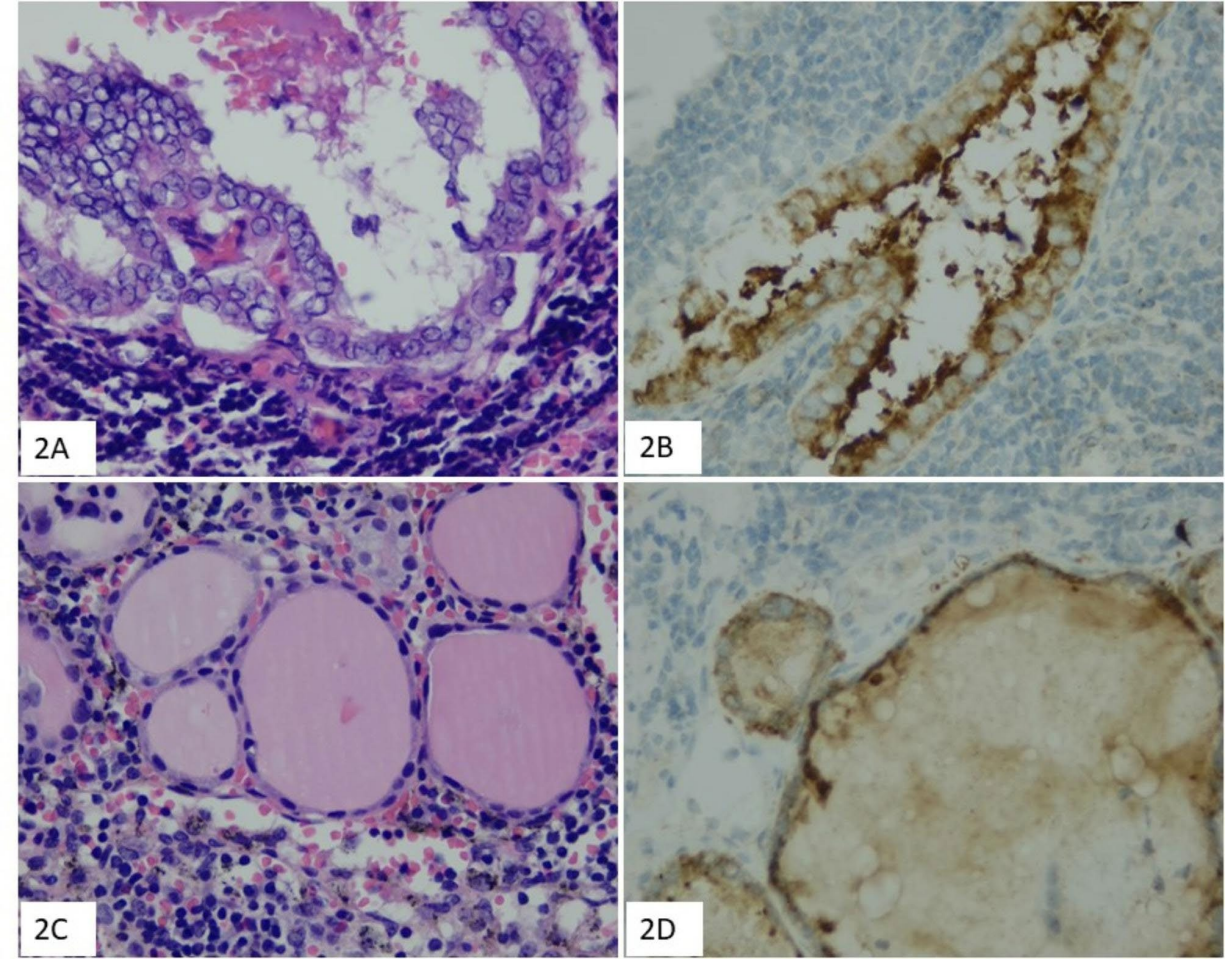
Incidentally detected papillary thyroid carcinoma in cervical lymph nodes, showing clear and crowded nuclei (2** A** HE×400), BRAF positive staining in cytoplasm of tumor cells (2**B** HE×400). Incidentally detected papillary thyroid carcinoma in cervical lymph nodes, showing follicular growth pattern and minimal nuclear atypia (2** C** HE×400), but BRAF positive staining in cytoplasm of tumor cells (2**D** IHC×400)

**Fig. 3:**
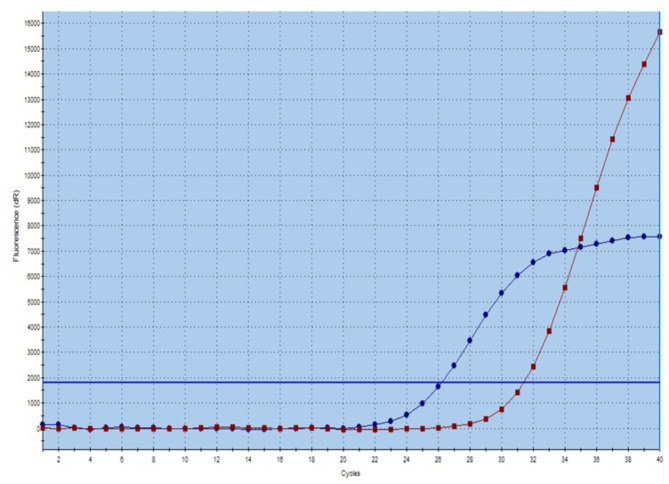
BRAF V600E mutation detection with ARMS PCR, representative curve of sample with mutant BRAF

A total of 57 lymph nodes were involved by PTC, of which 20 were level VI, 12 were level III, 10 were level II, 8 were level IV, 2 were level VII, and 5 were unknown location (Table 3). 11 patients presented ≥ 2 nodes involvement, and 20 patients presented only single node involvement. Papillary structure and nuclear feature were observed in 12 patients (Fig. 1). Psammoma bodies were found in 4 patients. One or several thyroid related markers (PAX8/TTF1/TG/CK19) were stained in most cases and confirmed thyroid origin. BRAF V600E immunohistochemical staining were performed in 17 metastatic lymph nodes with sufficient tumor tissues, and 6 were positive (Table 4; Fig. 2). BRAF V600E point mutation was detected in 5 of 6 BRAF V600E positive cases and was not detected in one BRAF V600E positive case (Fig. 3).

**Table 3:** Clinical levels of cervical lymph nodes involved by incidentally detected PTC

Clinical levels	N(%)
Level II	10(17.5%)
Level III	12(21.1%)
Level IV	8(14%)
Level V	0(0%)
Level VI	20(35.1%)
Level VII	2(3.5%)
Unknown	5(8.8%)

**Table 4:** Pathological characteristics of incidentally detected metastatic PTC

Clinical features	N(%)
**Levels of lymph nodes**	
Central	22(38.6%)
Lateral	30(52.6%)
Unknown	5(8.8%)
**Number of foci**	
Unifocal	20(64.5%)
Multifocal	11(35.5%)
**Structure of morphology**	
Papillary	10(45.5%)
Follicular	12(54.5%)
**Nuclear feature of PTC**	
Yes	12(54.5%)
No	10(45.5%)
**Psammoma bodies**	
Yes	4(18.2%)
No	18(81.8%)
**BRAF V600E staining**	
Positive	6(35.3%)
Negative	11 (64.7%)

Subsequent ultrasound of the thyroid showed no nodules or calcifications/benign nodules in 20 patients, and suspected malignant nodules in 5 patients. The thyroid imaging examination for 6 patients was not carried out, and the data cannot be found in our medical record. 12 patients underwent total thyroidectomy or ipsilateral lobectomy. The imaging and histopathology information for the 12 patients underwent surgery was presented in Supplementary Table 1. For the 12 patients, 2 showed suspicious malignant gross nodules and were confirmed to be PTC after suspicious nodule sampling. The largest diameter of PTC tumor was 10 mm in one patient, 5 mm in the other, The remaining 10 patients who had no suspicious malignant gross nodule were sampled the entire thyroid tissues, and found PTC in 4 patients, with the largest diameter less than 1 mm (Table 5). Considering the histologic subtype of all the 6 PTC patients, 4 were classic and 2 were follicular subtype. For the remaining 6 patients underwent surgery, 2 were diagnosed as follicular adenomas, 2 were nodular goiter, 1 was lymphocytic thyroiditis and 1 was normal thyroid tissues. The 19 patients without surgery were kept under active surveillance. The median time of follow-up was 14.85 months (ranging from 6 to 42 months). No one had any kind of recurrence of PTC.

**Table 5:** Thyroid status of primary tumors

Thyroid status	N(%)
**Imaging results**	
Positive	5(16.1%)
Negative	20(64.5%)
Unknown	6(19.4%)
**Thyroidectomy**	
Yes	12(38.7%)
No	19(61.3%)
**Primary thyroid PTC**	
Positive	6(50%)
Negative	6(50%)

Next, we divided the patients into two groups. One included 20 patients showing only single node involvement. In this group, BRAF staining was performed in 8 out of 20 patients, and 3 were BRAF positive. 3 patients were suspicious for malignancy in imaging finding, and 8 underwent surgery. On the other group, 11 patients were 2 or more node involvement. BRAF staining was performed in 9 patients, and 3 were positive. 2 patients were suspicious for malignancy in imaging finding, and 4 underwent surgery (Supplementary Table 2).

## Discussion

The incidental finding of thyroid tissue in cervical lymph nodes during neck dissections performed in nonthyroidal cancer is rare. The prevalence reported in several previous studies ranged from 0.3–1.6% [14–16]. We observed a prevalence of 1.2% (31/2650) in a large cohort of head and neck non-thyroid carcinoma. All the inclusions were found incidentally in the pathological examination of the neck dissection specimen, and no clinical history and suspicion of PTC was reported in clinical records. Among all the 57 involved lymph lodes, central neck level (level VI) was most frequently involved, accounting for 35.1%. It is not surprising, as level VI is also the most common site and the first echelon for metastasis of PTC. Of the 31 patients in our cohort, 27 were male and the median age was 62, indicating an older male predominance, which is very different from primary PTC that presents young female predominance. As for the initial non-thyroid tumors, 93.5% (29/31) were squamous cell carcinomas. These findings could be interpreted by the high incidence of squamous cell carcinomas in head and neck of males.

Although the incidental discovery of thyroid tissue in cervical lymph nodes is generally regarded as a metastatic lesion derived from thyroid gland, sometimes it remains controversial in the diagnosis between metastatic PTC and benign thyroid inclusions. It is not easy to determine metastases or benign heterotopia, especially for the morphologically benign appearing thyroid follicles in lymph nodes. As salivary glands, naevus cells and Müllerian epithelia aberrantly occurred in lymph nodes have been well-established, the possibility of benign thyroid heterotopia has also been proposed [17–19]. In addition, diagnostic criteria have been suggested for benign heterotopia in lymph nodes: (1) adjacent to or in the lymph node capsule, (2) no more than two nodes involvement, (3) morphology of thyroid follicles and absence of nuclear features of thyroid papillary carcinoma, (4) absence of psammoma bodies and desmoplastic stroma, (5) inconsistent immunohistochemical and molecular profiling for thyroid cancer [17, 18]. The diagnosis of benign heterotopia might be considered only when all the conditions mentioned above are fulfilled. However, some pathologists contest the opinion of benign heterotopia in lymph nodes as there is no confirmed embryological or anatomic evidence for such inclusions. Moreover, it is not uncommon that metastatic well-differentiated follicular variant of PTC presents normal-like appearance in cervical lymph nodes. In the context of molecular evidence, the BRAF V600E point mutation has been reported in morphologically benign appearing thyroid inclusions of cervical lymph nodes [20]. This finding would question the notion of benign nodal thyroid heterotopia. Taken together, occult metastatic carcinoma in cervical lymph nodes must always be taken into consideration even if histopathologic studies do not reveal features of PTC. In our present study, 22 cases were available for morphological evaluation, 10 showed normal-looking thyroid follicular morphology, and 12 presented at least one of the following features: papillary structure, nuclear features of PTC and psammoma bodies. Among the 10 cases with normal-looking thyroid follicular morphology, 2 underwent thyroidectomy and PTC was found after a thorough sampling. It has been generally believed that a primary PTC is undoubtedly necessary for the development of an occult metastatic thyroid carcinoma. Identification of primary PTC in the 2 postoperative thyroid specimens in our study indicates the possibility of an occult metastasis rather than an ectopia of benign appearing thyroid follicles in cervical lymph nodes. It must be mentioned that the two primary PTCs were considerably small (less than 1 mm in size). As most PTCs are not highly aggressive, it seems to be unusual for small tumors to metastasize. However, our observation emphasizes that no matter how small the tumor is, even less than 1 mm, it might have a significant potential for metastasis.

Subsequently, we performed BRAF V600E immunohistochemical staining in 17 cases with sufficient tumor cells, and 6 showed positive staining. Further BRAF V600E point mutation was detected in 5 of the 6 BRAF V600E positive cases. As there was no BRAF V600E point mutation in 1 IHC positive case, we reexamined the HE slides and found less than 10% tumor cell content in the nodes. Probably, the negative molecular result might be caused by the too small population of tumor cells. In our findings, the prevalence of BRAF V600E point mutation is slightly lower than that of primary thyroid origin PTC [7]. It is worth mentioning that one case showing bland-looking thyroid tissue was also positive for BRAF staining. This finding further supports the opinion of malignant PTC metastasis other than benign heterotopia.

When occult metastatic PTCs were identified in the cervical lymph nodes in the context of more aggressive squamous cell carcinomas, it is difficult to determine a single standard treatment as it should be based on overall survival of individual patients. Given the natural history of PTC, it is often less aggressive than the initial non-thyroid origin carcinomas of head and neck. Therefore, the prognosis of such patients usually depends on the more aggressive carcinomas but not PTC. Even so, evaluation of a thyroid tumor is also essential. When occult metastatic PTC was identified, imaging examination of thyroid should be carried out in routine clinical practice. Clinical management regarding surgery or surveillance hinges on both clinical and radiological features. In the cohort of 31 cases, imaging examinations were performed in 25 patients, among them, 5 showed suspicious malignant thyroid nodules and 20 were normal or benign nodules. 12 underwent total thyroidectomy or lobectomy, and pathological results revealed PTC in 6 patients after a thorough sampling. However, among the 6 patients displaying PTC, 4 were less than 1 mm in size. Moreover, PTCs were not identified in 50% (6/12) patients with surgery, suggesting that primary PTC of thyroid was not always identified in partial or total thyroid dissection specimen. Despite the negative thyroid finding of imaging or histopathology, the metastatic nature of the lymph node localization cannot be entirely excluded. The incidental discovery of PTCs in cervical lymph nodes, of course, is not always a metastasis but may occasionally be a primary lesion arising from heterotopic thyroid. On the other hand, although primary PTCs were found in 3 cases with benign imaging results, the lesions were considerably small. Therefore, if imaging shows normal or benign appearance, active surveillance could be recommended. However, when imaging reveals suspicious malignant thyroid nodules, the decision for thyroidectomy or conservative approach would be affected by multiple factors, including age, tumor size, pathological histological subtypes and clinical stage of initial non-thyroid carcinomas, as well as patients’ wishes. Our results reveal that the majority of patients do not necessarily perform aggressive surgical dissection considering the minimal influence on patient outcome. These observations are also in consistent with several previous studies [21, 22].

However, there is still some limitations in our study. Firstly, 9 out of 31 cases were not reevaluated for morphological analysis due to data withdrawn from consultation pathology and unavailable for original sections. Secondly, as the incidentally discovered metastatic PTCs are usually small, several cases are insufficient for the following molecular testing. Especially for the benign-looking lesions, further studies are still needed to clarify their malignancy based on a larger sample size and more profound molecular analysis.

## Conclusion

Incidentally discovered PTC in lymph nodes has usually interpreted as metastasis from a clinical occult thyroid primary papillary carcinoma, although primary PTC of thyroid was not always detected in partial or total thyroid dissection specimen. Our findings suggest it would be double occult lesions. Regarding concurrence with highly malignant non-thyroid tumor, the incidentally detected metastatic PTC alone does not seem to influence disease progression. Clinical management considering surgery or surveillance depends on clinical and radiological tumor features, and most patients could keep regular ultrasonic surveillance if further thyroid ultrasound does not show any malignant imaging sign.

## Electronic Supplementary Material

Below is the link to the electronic supplementary material.

Supplementary Material 1
