# Supplementary material for: Clinicopathological features of incidentally detected metastatic thyroid papillary carcinoma in cervical lymph nodes of non-thyroid cancer patients: a retrospective analysis of 31cases
Source: Diagn Pathol. 2023 Jul 15;18:82. doi: 10.1186/s13000-023-01370-4 (PMC10350260; doi:10.1186/s13000-023-01370-4)
Supplement: Supplementary file 1 — Supplementary Material 1 [file 13000_2023_1370_MOESM1_ESM.docx]

Supplementary table 1: Imaging and pathology information for 12 patients underwent surgery.

| Cases | Radiology Finding | Histopathology |
| --- | --- | --- |
| 1 | suspicious | PTC, 10mm |
| 2 | suspicious | PTC, 5mm |
| 3 | suspicious | PTC, <1mm |
| 4 | benign | PTC, <1mm |
| 5 | benign | PTC, <1mm |
| 6 | benign | PTC, <1mm |
| 7 | benign | Follicular adenoma |
| 8 | benign | Follicular adenoma |
| 9 | benign | nodular goiter |
| 10 | benign | nodular goiter |
| 11 | benign | lymphocytic thyroiditis |
| 12 | benign | normal thyroid tissues |

Supplementary table 2: Related information for single and multifocal node involvement.

|  | Unifocal | Multifocal |
| --- | --- | --- |
| Number | 20 | 11 |
| BRAF testing | 8 | 9 |
| BRAF positive | 3 | 3 |
| Suspicious nodules  (USG finding) | 3 | 2 |
| Surgery | 8 | 4 |
